# Use of additives to regulate solute aggregation and direct conformational polymorph nucleation of pimelic acid

**DOI:** 10.1107/S2052252521000063

**Published:** 2021-02-06

**Authors:** Peng Shi, Shijie Xu, Huaiyu Yang, Songgu Wu, Weiwei Tang, Jingkang Wang, Junbo Gong

**Affiliations:** a School of Chemical Engineering and Technology, State Key Laboratory of Chemical Engineering, Tianjin University, Tianjin 300072, People’s Republic of China; b The Co-Innovation Center of Chemistry and Chemical Engineering of Tianjin, Tianjin 300072, People’s Republic of China; c Tianjin Key Laboratory of Marine Resources and Chemistry, College of Chemical Engineering and Materials Science, Tianjin University of Science and Technology, Tianjin 300457, People’s Republic of China; d Department of Chemical Engineering, Loughborough University, Loughborough LE11 3TU, United Kingdom

**Keywords:** crystal nucleation, conformational polymorphs, additives, self-assembly, crystal engineering, intermolecular interactions, polymorphism, crystal growth, hydrogen bonding

## Abstract

Understanding the nucleation pathway and achieving regulation to produce the desired crystals are mutually beneficial. The authors previously proposed a nucleation pathway of conformational polymorphs in which solvation and solute self-assembly could affect the result of the conformational rearrangement and further nucleation outcomes. Based on this, herein α,ω-alkanedi­carb­oxy­lic acids (DA*n*, where *n* represents the number of carbon atoms in the molecule, *n* = 2–6, 8–11) were designed as homologous additives to interfere with the self-assembly of pimelic acid (DA7) to further induce the form II compound, which differs from form I only in conformation. Interestingly, longer-chain additives (DA6–11) have a stronger form II-inducing ability than short-chain ones (DA2–4). In addition, an apparent gradient of the degree of interference with solute self-assembly, consistent with form II-inducing ability, was detected by infrared and nuclear magnetic resonance spectroscopy. The calculated molecular electrostatic potential charges also clearly indicate that additive–solute electrostatic interactions gradually increase with increasing carbon chain length of the additives, reaching a maximum value with DA6–11. This novel use of additives demonstrates a direct link between solute aggregation and conformational polymorph nucleation.

## Abstract 2

The interference of solute self-assembly caused by the interactions between pimelic acid and a series of homologous additives is closely related to the ability to induce the form II compound with similar packing but a different conformation to that of form I. The novel use of additives demonstrates the direct link between solute aggregation in solution and molecular conformation in crystals.

## 1. Introduction   

From the viewpoint of crystal engineering and crystalline product quality, it is of great importance to control and predict crystal nucleation for crystal design and regulation (Desiraju, 2013 ▸; Bučar *et al.*, 2013 ▸). Over the last decades, significant progress has been made in understanding and regulating molecular pathways of solution nucleation of organic compounds (Davey *et al.*, 2013 ▸; Zeng *et al.*, 2018 ▸; Cruz-Cabeza *et al.*, 2017 ▸; Friščić & MacGillivray, 2009 ▸). On one hand, the structural relevance of solutes in solution and in the solid state was frequently used as a probe to uncover the structural process from molecule to crystal (Yang *et al.*, 2014 ▸). Multiple structural systems which can provide richer structural probes, such as polymorphs (Zeng *et al.*, 2018 ▸), co-crystals (Chadwick *et al.*, 2009 ▸) and solvates (Parveen *et al.*, 2005 ▸) were selected as research models for nucleation pathways. On the other hand, different methods have been employed to control crystal forms, such as changing solvent, supersaturation or temperature (Hansen *et al.*, 2016 ▸; Shi *et al.*, 2018 ▸), adding soluble additives (Li *et al.*, 2019 ▸; Weissbuch *et al.*, 2003 ▸) or insoluble templates (Yang *et al.*, 2017 ▸; Tulli *et al.*, 2014 ▸), or introducing laser light fields (Sun *et al.*, 2008 ▸; Rungsimanon *et al.*, 2010 ▸). This variety of approaches can be mutually beneficial. A deeper understanding of the crystallization mechanism will guide the regulation of desired crystals.

**Figure 1:**
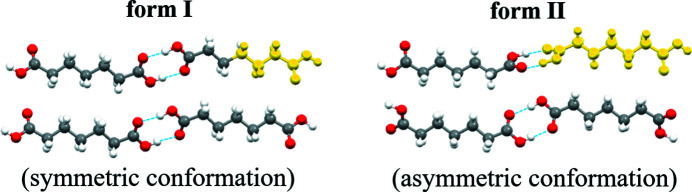
Crystal packing of the two forms. The fraction of the molecule in yellow is the asymmetric unit of two forms, respectively.

As for the most widely studied polymorphism, starting from 2,6-di­hydroxy­benzoic acid (Davey *et al.*, 2001 ▸), structural similarity between self-assembled aggregates or conformations in solution and molecular synthons in the resultant crystal were found in different cases, such as glycine (Tang *et al.*, 2017*a* ▸), tetrolic acid (Parveen *et al.*, 2005 ▸), isonicotinamide (Kulkarni *et al.*, 2012 ▸), *n*-phenyl­hydroxamic acid (Yamasaki *et al.*, 2006 ▸), 1,1,3,3,5,5-hexa­chloro-1,3,5-trigerma­cyclo­hexane (Ischenko *et al.*, 2005 ▸) and so forth. Meanwhile, in other examples (Back *et al.*, 2012 ▸; Du *et al.*, 2015 ▸; Davey *et al.*, 2013 ▸; Li *et al.*, 2020 ▸), more complicated restructuring can occur since no direct link between solute species in solution and crystal structure was found. Despite the controversy on this topic, to some extent, these studies suggested multiple possible nucleation pathways. In theory we could design and regulate nucleation outcomes at the molecular level according to the possible nucleation pathways. At present, some reported cases on controlling nucleation outcomes are based on synthon differences. Zeng *et al.* (2018 ▸) tune the polymorphic outcome of tetrolic acid and isonicotinamide by ionic liquids on the difference of strength between carboxyl (or amide) dimers and catemers. Similarly, the nucleation of pyrazinamide (Zhang *et al.*, 2018 ▸), isonicotinamide (Caridi *et al.*, 2014 ▸; Kulkarni *et al.*, 2014 ▸) and 2,6-di­hydroxy­benzoic acid (Kulkarni *et al.*, 2014 ▸) could be controlled by templates according to the synthon difference. By comparison, structural pathways of conformational polymorph formation have less been verified and applied to crystallization control. In our previous work (Shi *et al.*, 2020 ▸), a possible structural pathway of conformational polymorph nucleation was proposed by comparing a series of α,ω-alkanedi­carb­oxy­lic acids (DA*n*). Their common dimorphs have similar packing patterns (the basic and unique synthon is a carboxylic acid dimer) with a difference in molecular conformations, DA7 is given as an example in Fig. 1 ▸. Their polymorphic outcomes, with the exception of DA5, show solvent dependence: form I with conformation I crystallizes from solvents with hydrogen-bond donating (HBD) ability, whereas form II with conformation II crystallizes preferentially from solvents with no HBD ability. In contrast, form II of DA5 does not crystallize in any of the solvents used. By combining spectroscopic analysis and computational simulation, we proposed the possible nucleation pathway: solvation and solute self-assembly has a remarkable effect on the result of conformational rearrangement and nucleation outcome. This indicates that we might be able to control the polymorphic outcomes by altering and interfering with solute aggregation configuration in solution. If conformational polymorphic outcomes could be tuned by molecular regulation, not only the proposed relatively complicated pathway would be further verified, but a novel approach to nucleation control may also be developed, especially for conformational polymorphs.

**Figure 2:**
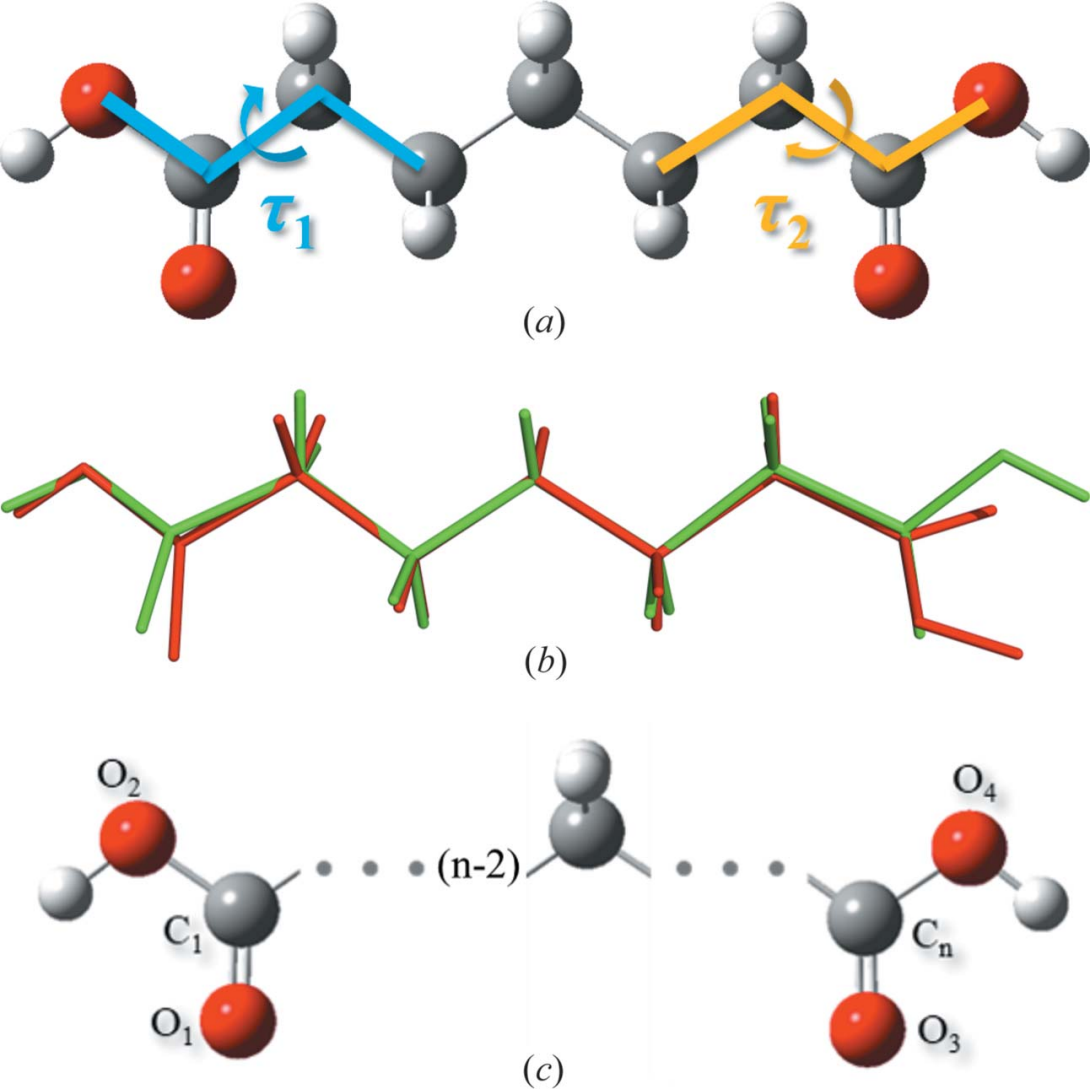
(*a*) Chemical structure of pimelic acid (DA7); the torsions at both ends of the molecule are defined as τ_1_ and τ_2_. (*b*) Molecular conformations of form I (green) and form II (red) for comparison; τ_1_ and τ_2_ are the main differences between the two forms. (*c*) Schematic of chemical structures of the diacids as additives with different numbers of carbon atoms, *n* = 2, 3, 4, 5, 6, 8, 9, 10, 11.

Herein pimelic acid (DA7) was selected as a representative research model owing to its high solubility which benefits experimental design. There are 16 refcodes of DA7 in the Cambridge Structural Database. These refer to forms beta and alpha, named I and II here and in our previous work for the purpose of unified discussion of the series. In fact, pimelic acid exists in three different polymorphic forms (Burger *et al.*, 1996 ▸; Cooke *et al.*, 2010 ▸). The crystal structure of the other form is unknown and rarely mentioned. Here we used 2 of the 16 crystal structures with small *R* values (CCDC Nos. 1233866 and 929796). The conformation of form I (CCDC No. 1233866) (Thalladi *et al.*, 2000 ▸) is molecular symmetry related with τ_1_ = τ_2_ = ±162.99° at both ends [Figs. 2 ▸(*a*) and 2(*b*)]. However, in the form II conformation, only one carboxyl has a sharp twist about τ_2_ = ±37.01° (τ_1_ = ±176.55°) (CCDC No. 929796) (Bhattacharya *et al.*, 2013 ▸), leading to a loss of molecular symmetry (Fig. 1 ▸). 1,4-dioxane was chosen as the solvent in this work, in which solute aggregates can form in abundance and stable form I is preferred (Shi *et al.*, 2020 ▸). Previous works (Li *et al.*, 2019 ▸, 2020 ▸; Dowling *et al.*, 2010 ▸; Weissbuch *et al.*, 2003 ▸) reported that the effects of additives on polymorph formation were mainly induced by modifying the growth of certain polymorphs. Davey *et al.* (1997 ▸) reported the rare case of polymorph control by using additives chosen on the basis of conformation. In this work we take a material which appears to self-aggregate in solution and we attempt to use additives to interfere with this aggregation to regulate conformational polymorph nucleation. Other α,ω-alkanedi­carb­oxy­lic acids were taken into account since they are more likely to be disruptive in solution as similar homologous compounds. Therefore, we attempted to use nine diacids (DA2/3/4/5/6/8/9/10/11) [Fig. 2 ▸(*c*)] as additives in the present work.

## 2. Discussion   

**Figure 3:**
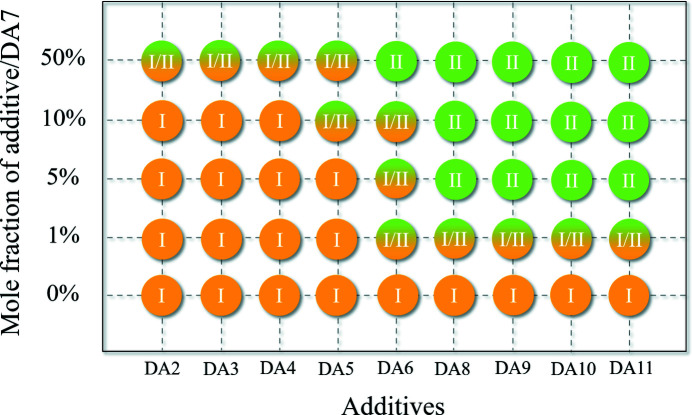
Crystallization outcomes of DA7 obtained in the presence of different additives with different concentrations.

Firstly, cooling experiments were conducted with the same initial concentration of DA7 in dioxane with the addition of 9 additives in the molar ratio 1:10 of additive to DA7 (details are given in the supporting information). Interestingly, different crystal forms of DA7 were obtained [confirmed by powder X-ray diffraction (PXRD), Fig. S1 of the supporting information] with additives of different carbon chain lengths (Fig. 3 ▸). Form I was obtained without any additives, as well as with 10 mol% of DA2–DA4. With longer carbon chain additives, such as DA5 and DA6, a mixture of form I and II appeared, and pure form II was obtained with DA8–DA11. To clarify the boundary conditions for polymorph formation, a series of additive concentrations (molar ratio 1:100, 5:100, 50:100 of additive to DA7) were applied in cooling crystallization. The results are summarized in Fig. 3 ▸ (PXRDs are shown in Figs. S2–S10). At each additive concentration, additives with longer carbon chains have a greater ability to induce form II than those with shorter chains, and DA5 and DA6 seem to be transition points. Furthermore, with only 1 mol% additive of DA–DA11, some metastable II crystals formed. With DA2–DA5 as the additives at high percentage (50%), only mixtures can be produced. Thus the target of inducing form II was successful, which supported our hypothesis. Interestingly, an obvious difference in the ability of additives to induce form II was identified. In addition, similar cooling experiments of DA9 were conducted (DA2/3/4/5/6/7/8/10/11 as additives at the concentration of the molar ratio 1:10 of additive to DA9). From the results we can clearly see that the same trends hold true and DA7 is not the exception (Fig. S11).

In fact, in the cooling experiments, the nucleation temperature and supersaturation (*S*,*S* = *C*/*C*_s_, where *C* is the actual concentration of solute and *C*_s_ is the solubility of the solute) were not fixed, which influenced polymorph formation. Hence, solubility of DA7 on addition of other diacids should be measured to investigate the change of supersaturation, which may cause nucleation of different polymorphs. We took DA3, DA5, DA9 and DA11 as representatives based on the above result. A static gravimetric method (details are given in the supporting information) was adopted to measure the solubility of DA7 with and without four additives in 1,4-dioxane (0.1464 mol additive:mol solvent, equal to the molar ratio 5:100 of additive to DA7 in the above experiments) at 298.15 K. Additives have played the role of solubilization and the longer the carbon chain, the greater the effect (Fig. S11).

To eliminate the influence of supersaturation and temperature in previous experiments, two groups of isothermal crystallization experiments at *T* = 298.15 K with and without additives were carried out (details are given in the supporting information): group 1 was carried out with constant supersaturation (*S* = 1.5 with and without additives) but a different concentration of DA7; group 2 was carried out with constant concentration but different supersaturation (*S* = 1.5 without additives only) of DA7. The nucleation outcomes are consistent with the above cooling experiments (Table 1 ▸). With additives DA9 and DA11 in solution, pure form II was harvested. Mixtures were obtained from DA5 solutions. Pure form I was produced without additive and with DA3. Therefore, we excluded these important factors which may influence nucleation outcomes and confirmed the important role of this series of additives.

**Figure 4:**
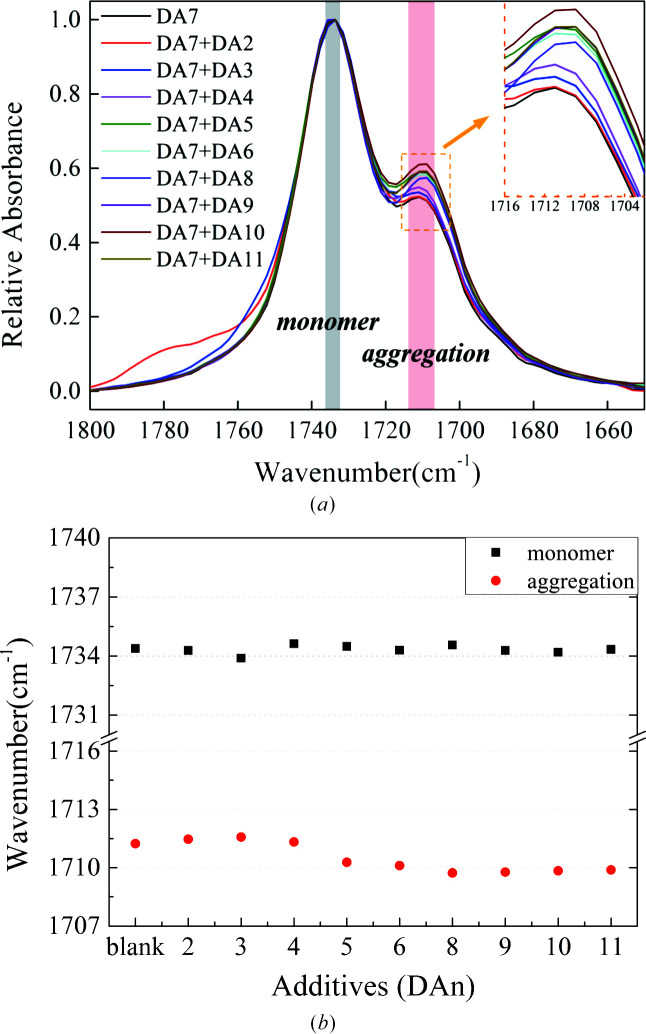
(*a*) Normalized FTIR spectra of solutions at a concentration of 1.5 mol l^−1^ solute + 0.25 mol l^−1^ additive. (*b*) Solution IR vibration peak position of C=O picked from (*a*) using the *OMNIC* software (DA*n*: *n* is the number of carbon atoms in the molecule).

According to previous work (Shi *et al.*, 2020 ▸), solute aggregations of DA7 in 1,4-dioxane were detected by variable concentrations of solution Fourier transform infrared (FTIR) spectra (Fig. S12). With increasing solute concentration, the vibration peak at about 1712 cm^−1^ appears and grows gradually, which indicates strong carboxyl–carboxyl interactions between solute molecules (Parveen *et al.*, 2005 ▸; Khamar *et al.*, 2014 ▸; Kulkarni *et al.*, 2012 ▸). Herein, we attempted to monitor the response of IR spectra when various additives were added to DA7 solutions at a uniform concentration. Considering possible, more-significant responses, a relatively high concentration of 0.25 mol additive per litre of solvent was added to the DA7 solutions with a concentration of 1.5 mol l^−1^ (solute/solvent), including DA2–DA6 and DA8–DA11. As expected, the peak intensity of solute aggregation at about 1712 cm^−1^ increases in all samples studied (Fig. 4 ▸). This means more carboxyl–carboxyl interactions occur, which might be solute–additive, additive–additive or both. As a comparison, there is no obvious peak at a similar position in the spectra of pure additives dissolved in dioxane at the same concentration of 0.25 mol l^−1^ (additive/solvent) [Fig. S13(*a*)]. Hence, interactions between solute DA7 and additives are very likely to form. Surprisingly, from DA2 to DA4, their peak positions picked by *OMNIC* software, 1711.3–1711.7 cm^−1^, are almost consistent with those of no additives. As for DA5 and DA6, a small red shift is observed (1710.5 and 1710.3 cm^−1^). For DA8, DA9, DA10 and DA11, the peak position is almost at 1709.9–1710.2 cm^−1^. It is noted that the vibration peak position of carboxyl monomers at about 1734 cm^−1^ remains essentially unchanged without obvious shift. The red shift of the vibration peaks of carboxyl aggregations of longer-chain additive molecules indicates that the intermolecular interactions are stronger.

**Figure 5:**
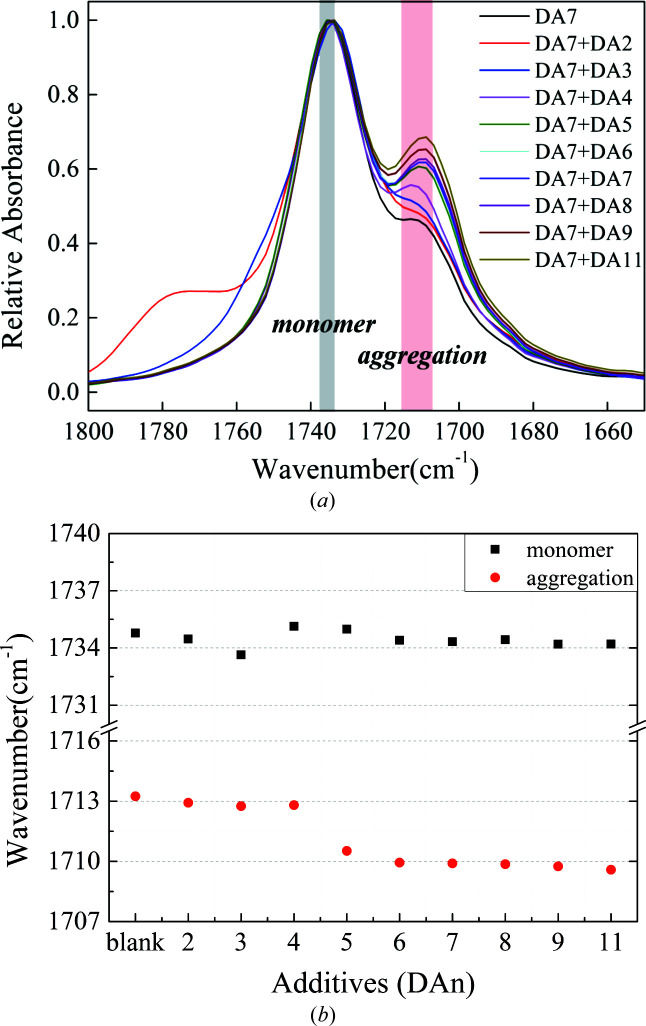
Normalized IR spectra of solutions at a concentration of 1.2 mol l^−1^ solute + 0.6 mol l^−1^ additive. (*b*) Solution IR vibration peak position of C=O picked from (*a*) using the *OMNIC* software (4 cm^−1^ resolution).

However, we realized that this was not a rigorous and reliable scientific result because a resolution of 4 cm^−1^ was used in these tests, which is larger than the reported red shift. To further verify the signals, a higher additive concentration of 0.6 mol l^−1^ was added to DA7 solutions to produce a final solute concentration of 1.2 mol l^−1^. An equivalent amount of DA7 was added as the blank. The amount of additive was increased while that of solutes was simultaneously decreased in order to maintain easy dissolution and improve their molar ratio to amplify the response (chemical shift). In addition, we utilized resolutions of 2 cm^−1^ (as small as possible) as well as 4 cm^−1^ in the analysis (Figs. 5 ▸ and S14). The new results show a similar and more pronounced trend: the red shift of the vibration peak of carboxyl aggregations from DA2–4 to DA8–11 reached about 3 cm^−1^, and DA5 was still in transition. It was a strong signal characteristic of aggregation, supporting the occurrence of solute–additive interactions. Although slight aggregation existed in pure additive solution at this concentration [Fig. S13(*b*)], when acting as additives, the peak intensity of solute aggregation showed an obvious increase. More importantly, the peak positions of aggregation while adding these additives were similar to those of DA7 solute added in the same amount [Fig. 5 ▸(*b*)], distinct from the additive self-aggregation position (at a higher wavenumber) at this concentration. This suggests that these additive molecules were indeed involved in DA7 solute self-assembly. Furthermore, we found that the resolutions used had few effects on the test results in terms of chemical shift, indicating that the results were reliable. In our previous work (Shi *et al.*, 2020 ▸), we attempted to influence polymorph nucleation using benzoic acid. No changes in crystal nucleation were observed when adding benzoic acid even at high concentration. There was no obvious signal in the solution FTIR spectra to show whether benzoic acid can disturb the intermolecular interactions in DA7 solution. This still supports the results and conclusions here. In brief, with a variety of additives, an apparent gradient of interactions between solutes and additives can be observed.

**Figure 6:**
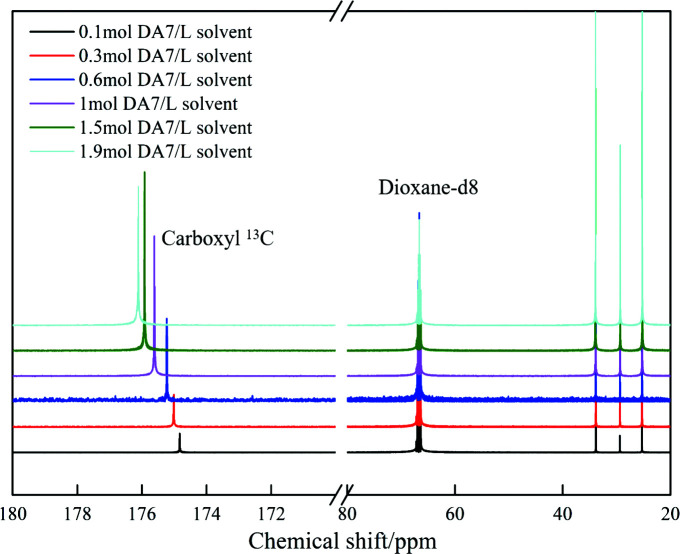
NMR spectra of pure DA7 over a concentration range in dioxane-d8.

**Figure 7:**
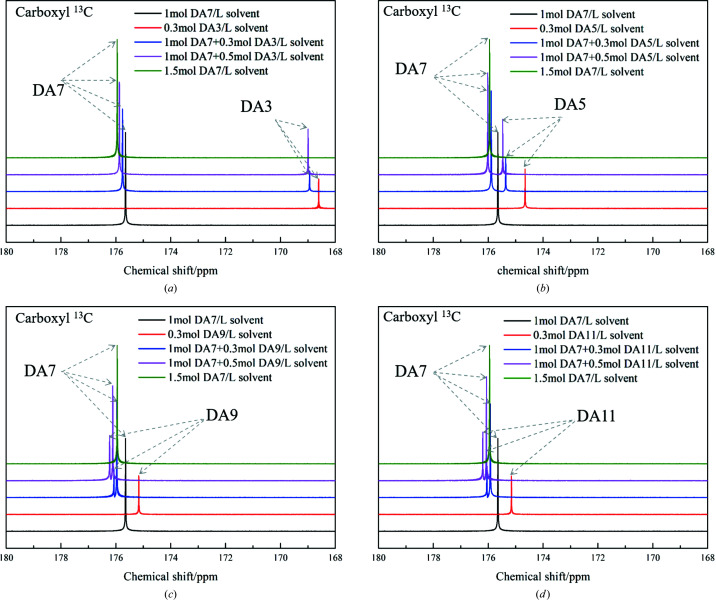
Carboxyl ^13^C NMR spectra of solutions of solute/solute + additives/additives in dioxane-d8: (*a*) DA7 with DA3; (*b*) DA7 with DA5; (*c*) DA7 with DA9; (*d*) DA7 with DA11.

Intermolecular interactions between additives and solutes in solution were further investigated by measuring ^13^C chemical shifts. The chemical shifts reflect the ensemble-averaged interactions in solution but, at the same time, are highly sensitive to subtle changes in the local chemical environment of a molecule (Tang *et al.*, 2017*b* ▸,*c* ▸). The carboxyl ^13^C chemical shift shown in Fig. 6 ▸ displays a remarkable downfield trend when the concentration of DA7 increases. The deshielding of ^13^C resonance implies the formation of hydrogen bonding between carboxyl groups of DA7 molecules (Tang *et al.*, 2017*b* ▸,*c* ▸), echoing the FTIR finding. The ^13^C NMR experiments of DA7 with additives (still taking DA3/5/9/11 as examples) in 1,4-dioxane-d8 were also conducted (Fig. 7 ▸). When DA3 at a concentration of 0.3 mol l^−1^ was added to DA7 solution at 1 mol l^−1^ (solute/solvent), the NMR peaks (chemical shifts) of the DA7 carboxyl ^13^C shift slightly downfield from δ = 175.65 (pure DA7 at a concentration of 1 mol l^−1^) to 175.77 p.p.m. And that of DA3 carboxyl ^13^C shifts downfield from δ = 168.58 (pure DA3 at a concentration of 0.3 mol l^−1^) to 168.95 p.p.m. This demonstrates that interactions between carboxyl groups of DA3 and DA7 exist. Moreover, when the same concentration of longer-chain diacids (DA5 or DA9) was added, more chemical shifts of the carboxyl ^13^C of DA7 and additives happen, respectively. It is clear that, within limits, the longer the carbon chain of the additive, the greater the number of and the stronger the hydrogen bonds between solute and additive molecules are. Interestingly, when DA11 is the additive, the NMR peaks (chemical shifts) of the carboxyl ^13^C of solute and additive remain fairly consistent with those of DA9. This suggests that interference effects of additives with longer chains on solute aggregation are similar, corresponding with the FTIR finding and the nucleation results. When more additives (at a concentration of 0.5 mol l^−1^) are added to the DA7 solution at 1 mol l^−1^, more shifts of the carboxyl ^13^C of DA7 and additives are observed. These make it clear that increasing the concentration of additives will increase the interactions between the solute and the additives. Furthermore, from DA3 (δ = 175.88), DA5 (δ = 176.00) to DA9 (δ = 176.12), the carboxyl ^13^C of DA7 in these solutions (1 mol l^−1^ solute + 0.5 mol l^−1^ additive) shift close to that of pure DA7 at 1.5 mol l^−1^ (δ = 175.95). When DA9 (δ = 176.12) and DA11 (δ = 176.10) are used as additives, the peak of the carboxyl ^13^C of DA7 appears even further downfield than that of pure DA7 (δ = 175.95). NMR data strongly illustrate the roles of additives as modifiers of solution aggregation.

**Figure 8:**
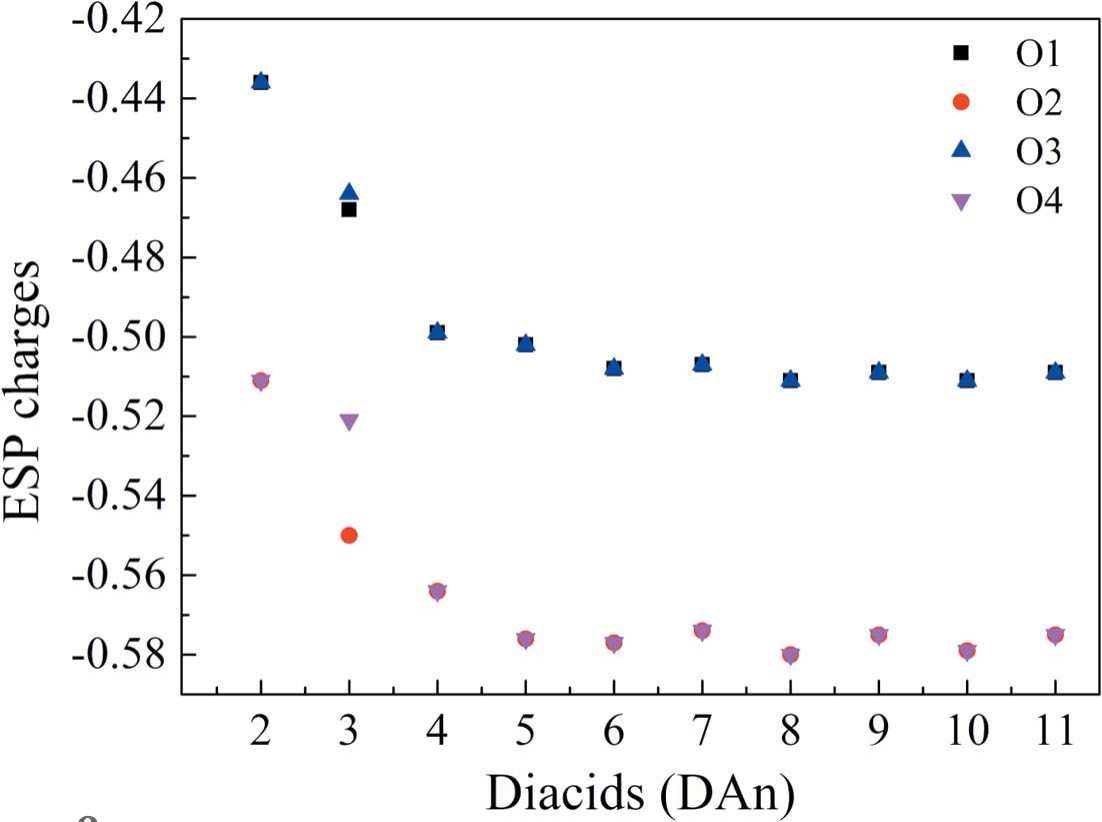
ESP charges on all oxygen atoms of solute and additives. The number of oxygen atoms are shown in Fig. 2 ▸(*c*) (DA*n*: *n* is the number of carbon atoms in the molecule).

In order to further understand this gradient of solute–additive interactions, the molecular electrostatic potential (ESP) charges (Murray & Politzer, 2017 ▸) of the oxygen atoms in COOH groups of additives were calculated at DFT levels, as an indicator of the electrostatic interaction between the additives and solutes (Wang *et al.*, 2019 ▸) (details are provided in the supporting information). The simulation results (Fig. 8 ▸) indicate a gradual increase in ESP charges of all oxygen atoms from DA2 to DA5, up to DA8–DA11 whose charges are equivalent to that of DA7. These indicate that additive–solute electrostatic interactions gradually increase with increasing carbon chain length of the additives, reaching a maximum value with DA8–DA11, then remaining equal to solute–solute interactions. Thus, these additives appear to have a stronger interference with solute self-assembly, which is consistent with intermolecular interactions detected by solution IR and NMR, and is also reflected by the polymorph nucleation outcomes.

Previous research (Li *et al.*, 2019 ▸; Liu *et al.*, 2020 ▸; Dowling *et al.*, 2010 ▸; Weissbuch *et al.*, 2003 ▸) reported that the effects of additives on polymorph formation were mainly induced by modifying the growth. Here we have also investigated the influence of additives on the crystal shape and growth. The results (Fig. S16) show that when a large number of form II crystals are produced, induced by additives, the growth of form I remains unchanged and is not dramatically influenced. This reflects the main effects of additives on polymorphic nucleation rather than crystal growth. In fact, for two such polymorphic structures with extremely similar stacking and non-polar crystals, it is unlikely for additives to completely inhibit or modify growth of only one of them, which is why we started with this material.

Thus the proposed nucleation pathway of this series of conformational polymorphs has been verified and successfully used to design additives to direct polymorph formation. The assembly of two crystal forms of DA7 is very similar. The basic and unique synthon is the carboxylic acid dimer. Assembled molecular chains aggregate into a layer, and then into a crystal through hydro­phobic interactions among the alkyl chains. In order to pack as close as possible and adjust the distance between carboxyl dimers to reduce in-plane O–O repulsions and keep the structure stable, the whole conformation twists in both forms, starting with carboxyl groups; twisting of other torsions is negligible (Shi *et al.*, 2018 ▸, 2020 ▸; Thalladi *et al.*, 2000 ▸). That is, despite the important role of the alkyl chains in crystal packing of chains and layers (Ma *et al.*, 2016 ▸; Bond, 2004 ▸), polymorph formation is in fact derived from the assembly of the carboxyl groups. Combined with the calculation of the relative stability of the conformations (Shi *et al.*, 2020 ▸), the final conformations of the two polymorphs are formed via chain-by-chain and layer-by-layer assembly during the nucleation process. According to our previous work (Shi *et al.*, 2020 ▸), conformation rearrangement is likely to occur in two steps: first to metastable conformation II, then to stable conformation I. Desolvation and solute aggregation are obstacles that must be overcome before these steps. In 1,4-dioxane with no HBDs, weak solvation results in solute desolvation and self-aggregation earlier, and the conformation sufficiently rearranges to stable form I. Herein the stronger interactions between the additive and DA7 are considered to be stronger ‘solvation’ interfering with solute self-assembly. Therefore, form II is favored to crystallize after insufficient conformation rearrangement when there exists stronger interactions between the additives and DA7.

## 3. Summary   

This work has demonstrated a good correlation between solute aggregation disturbed by a series of designed additives and conformational polymorph nucleation. Compared with the previous synthon link between the solution and solid structures, herein a proposed nucleation pathway for conformational polymorphs was applied and further verified from the perspective of crystal engineering. Meanwhile, novel use of additives was successfully employed to regulate the nucleation of conformational polymorphs with similar packing, which differed from their previous use of affecting crystal growth. More importantly, for the first time, this study has created a clear picture of the existing strong correlations between solute aggregation configuration in solution and molecular conformations in the crystal. Therefore, this contribution has shed light on how to directly control the conformational polymorph formation with the design of specific factors and we believe this rationale will have a bright future in other systems of interest.

## Supplementary Material

Supporting information file. DOI: 10.1107/S2052252521000063/lq5033sup1.pdf

## Figures and Tables

**Table 1:** Nucleation outcomes of DA7 obtained from non-thermal crystallization

Forms	Blank	DA3	DA5	DA9	DA11
Group 1†	I	I	I+II	II	II
Group 2‡	I	I	I+II	II	II

† The supersaturation of each batch was fixed at 1.5 based on thermodynamic data.

‡ The absolute concentration of DA7 was kept equivalent to the amount when *S* = 1.5 without additives.
