# Supplementary material for: Use of additives to regulate solute aggregation and direct conformational polymorph nucleation of pimelic acid
Source: IUCrJ. 2021 Feb 6;8(Pt 2):161–7. doi: 10.1107/S2052252521000063 (PMC7924234; doi:10.1107/S2052252521000063)
Supplement: Supplementary file 1 [file m-08-00161-sup1.pdf]

IUCrJ

Volume 8 (2021)

Supporting information for article:

Use of additives to regulate solute aggregation and direct conformational polymorph nucleation of pimelic acid

Peng Shi, Shijie Xu, Huaiyu Yang, Songgu Wu, Weiwei Tang, Jingkang Wang and Junbo Gong

S1. Supplementary experimental section

S1.1. Powder X-ray diffraction (PXRD)

PXRD data in this study were obtained on a Rigaku D/MAX 2500 X-ray diffractometer (Rigaku, Japan) by utilizing Cu-Kα radiation (λ=1.54178 Å) at 100 mA and 40 kV, which is used to determine crystalline forms of solid samples. Data were acquired at ambient temperature (298.15 K). Samples were scanned in the 2θ range of 5-30° with a scan speed of 8°/min in reflection mode.

S1.2. Fourier transform infrared spectroscopy (FTIR) and Nuclear magnetic resonance (NMR) analysis

FTIR spectra were determined for liquid samples on a Bruker FT-IR 750 spectrometer at ambient conditions. The wavenumber range is from 4000 to 400 cm^{-1} and the resolution is 2 cm^{-1} or 4 cm^{-1}. The background, the corresponding solvent to liquid samples was deducted in collecting spectrograms of samples. NMR spectra were detected using a 600 MHz JEOL JNM-ECZ600R/S1.

S1.3. Solubility measurement of DA7 with additives in 1, 4-dioxane

A gravimetric method was employed to determine the solubility of form I of DA7 with or without DA3/5/9/11 in 1, 4-dioxane (0.1464 mol additive / mol solvent, equal to the molar ratio 5:100 of additive to DA7 in above experiments) at 298.15 K. In this process, additive solutions in 1, 4-dioxane at the concentration of 0.1464 mol additive / mol solvent were prepared firstly. Then excess form I of DA7 and corresponding additive solutions, were added to 50 mL flasks so that to obtain the suspensions. Then the suspensions were shaken by a thermostatic bath shaker (CHY1015, Shanghai Sunny Hengping Scientific Instrument Co. Ltd., China) at a certain temperature under uncertainty of 0.1 K. And this process would last for 12 h which had been proved to be long enough to achieve solid-liquid equilibrium in preliminary experiment. After turning off the bath shaker, 5 mL of the supernatant liquor was filtered by the pre-cooled/heated syringes filters (0.22 µm) and moved into pre-weighted glass dishes as quickly as possible. Immediately, the total weight was determined. After that, the dishes were dried in a vacuum oven (DZ-2BC, Tianjin Taisite Instrument Co. Ltd., China) at T=343.15 K and their mass was periodically measured until the data remained constant, which meant that the solvent had been completely evaporated. In all above experiments, the masses were determined by an electronic balance (AB204-N, Mettler-Toledo, Switzerland) with an accuracy of ± 0.0001 g. The experiment was repeated three times for error reduction, and the result was from the average value.

The mole fraction solubility of DA7 (x_1) was calculated by using Eq. (S1-2):

$$m_1 = m_s - m_2/M_2 \times 0.1464 \times M_{add} \quad \text{(S1)}$$

$$x_1 = \frac{m_1/M_1}{m_1/M_1 + m_2/M_2} \quad \text{(S2)}$$

Where m_s represents the mass of solid, m_1 represents the mass of solute DA7, m_2 mean the masses of solvents. M_1, M_2 and M_{add} are the molecule mass of DA7, solvent and additives, respectively.

S1.4. Cooling and isothermal crystallization experiments with or without additives

S1.4.1. Cooling experiments

Cooling experiments were conducted with the initial concentration of DA7 in dioxane of 0.527 g/ml (0.01 mol DA7 / 3 ml dioxane) with adding 9 additives in different additive concentrations (the molar ratio 1: 100, 5:100, 50: 100 of additive to DA7). A given mass of DA7 form I and additives were added into 3 ml solvent and heated to 333.15 K to ensure solid was dissolved completely. Then the solutions were filtered through a preheated 0.22 μm syringe filter, and transferred into a jacketed vessel and held at 333.15 K for 30 min. After that the solution systems were cooled to 288.15 K with a cooling rate of 0.2 K/min while stirring (400 rpm) ~~by a magnetic stirrer~~. A blank experiment was carried out without additives at the same condition (stable form I obtained). The obtained solid, analyzed by powder X-ray diffraction (PXRD) to identify the form (**Figure S1**), was separated as soon as possible after nucleation during cooling to avert the underlying polymorphic transition (the transformation of pure form II in solution occur after 42 min since nucleation at least and that of mixtures of form I and II occur earlier (about 25 min since nucleation with high fraction of form I)). The samples used to observe crystal shapes were taken out after 20 min since nucleation before phase transition.

Cooling experiments were conducted with the initial concentration of DA9 in dioxane of 0.439 g/ml (0.007 mol DA9 / 3 ml dioxane) with adding 9 additives in different additive concentrations (the molar ratio 1: 10 of additive to DA7). A given mass of DA7 form I and additives were added into 3 ml solvent and heated to 333.15 K to ensure solid was dissolved completely. Then the solutions were filtered through a preheated 0.22 μm syringe filter, and transferred into a jacketed vessel and held at 333.15 K for 30 min. After that the solution systems were cooled to 288.15 K with a cooling rate of 0.2 K/min while stirring (400 rpm) by a magnetic stirrer. A blank experiment was carried out without additives at the same condition (stable form I obtained). The obtained solid, analyzed by powder X-ray diffraction (PXRD) to identify the form, was separated as soon as possible after nucleation during cooling to avert the underlying polymorphic transition.

S1.4.2. Isothermal experiments

To eliminate the influence of supersaturation and temperature in previous experiments, two groups of isothermal crystallization experiments at T=298.15 K with or without additives were carried out. In Group 1, the supersaturation of each batch was fixed at 1.5 based on thermodynamic data. In Group 2,

we kept the absolute concentration of DA7 equivalent to the amount when S=1.5 without additives so that solutes and additives are in the same molecular proportion. Based on this, given masses of form I of DA7 and corresponding additives (DA3/5/9/11, 0.1464 mol additive/mol solvent) were added into 10 g pure 1, 4-dioxane based on the desired concentration at 298.15 K, and heated to elevated temperature (348.15 K) to make the solute dissolved completely. Then the heated solutions were filtered through a preheated 0.22 μm syringe filter and simultaneously transferred into a glass test tube and held at 298.15 K for 30 min. After that the tubes were rapidly transferred into a thermostatic water bath (model 501 A, Shanghai Laboratory Instrument Works Co., Ltd., China) with an accuracy of ± 0.05 K while agitating (400 rpm) by a magnetic stirrer. After the induction stage of nucleation, the solid appeared. Then they would be separated from the suspension as soon as possible and analyzed by PXRD to determine the crystal form. Each experiment was repeated three times.

S1.5. Calculation of electrostatic potential (ESP) charges

In this work, the ESP maps and ESP charges of additives were calculated at DFT level in DMol3 module using the molecular modeling software package Materials Studio (Accelrys Inc., USA). Before analysis of ESP charges, the geometry optimization of molecules were performed at first by double numerical plus polarization (DNP) basic set. The Perdew-burke-ernzerhof (PBE) and generalized gradient approximation (GGA) were selected. The quality of self-consistent-field (SCF) tolerance was fine. The ESP charges were assigned on molecules based on analysis function in Dmol3 module. The calculations were carried out for two times for comparison: the structures of all molecules were generated manually or picked from crystal structures. It was shown that the results were little to do with the molecular source.

S1.6. Calculation of binding energies of DA7 with additives

Density functional theory (DFT) calculations have been applied using a Gaussian 09 package to investigate interactions in (1:1) molecular complexes of DA7 with additives in SMD solvent model (dioxane). Similarly with the Reference you provided, the equilibrium geometries of the modeled associates are calculated with a B97D Grimme's functional, which includes a long-range dispersion correction. This allows for better description of the van der Waals interactions and gives proper geometries of molecular clusters. Binding energies of the complexes are calculated using a double hybrid B2PLYPD functional, which combines exact Hartree−Fock exchange with an MP2-like correlation and long-range dispersion corrections. A Gaussian-type 6-31G (d,p) basis set is used for geometry optimization and a triple-ζ valence quality (TZVP) basis set is used for energy calculations of the (1:1) associates. Basis set superposition error (BSSE) is calculated in the gas phase for these systems to correct overestimation in binding energies due to the overlapping of basis functions.

S2. PXRD patterns of form I/II and samples of DA7 or DA9 crystallized in presence of series additives with different amounts in 1, 4-dioxane.

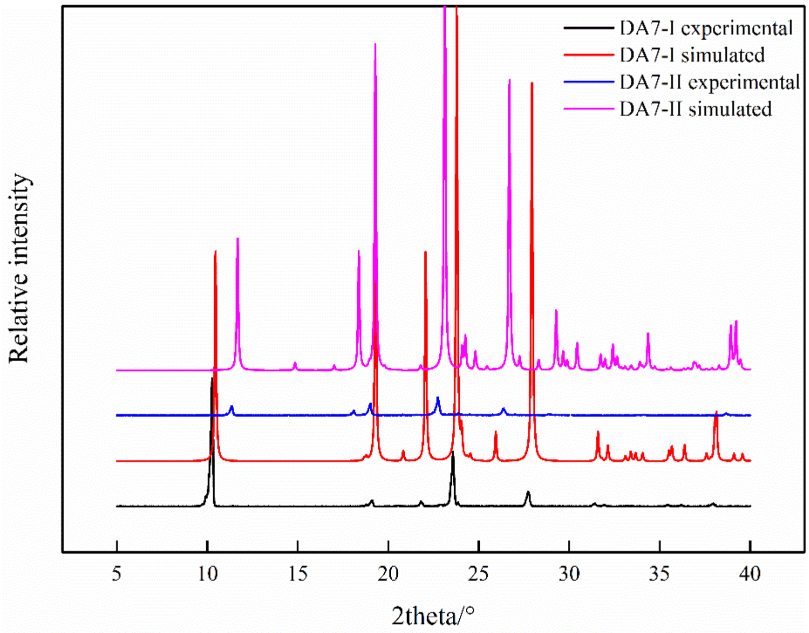

Figure S1 The experimental and simulated PXRD patterns of two forms of DA7.

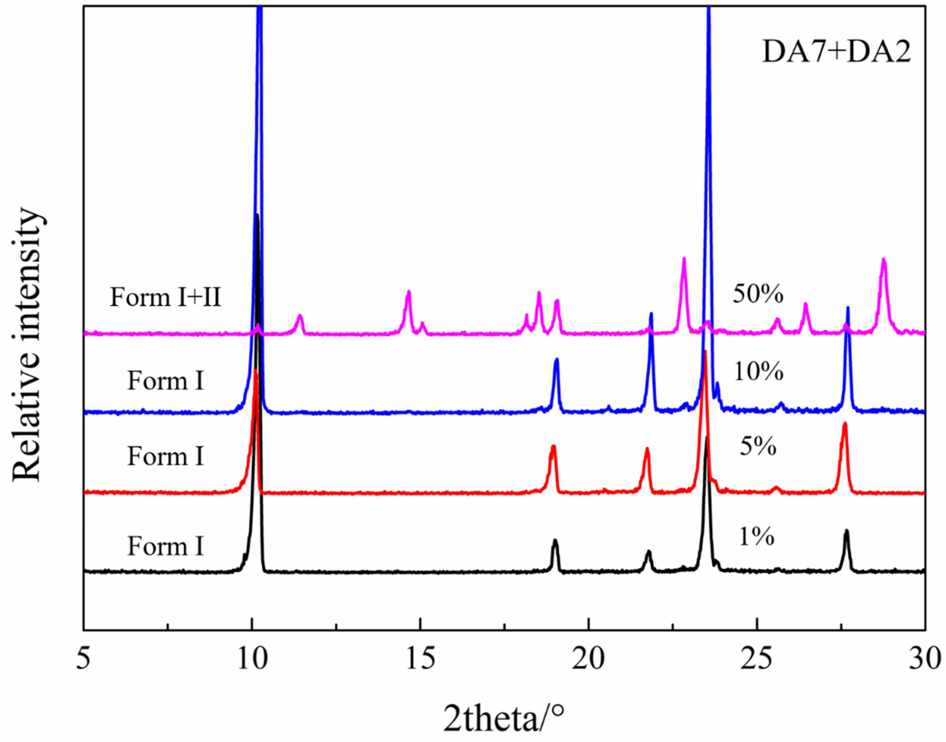

Figure S2 PXRD patterns of DA7 crystallized in presence of DA2 with different amounts in 1, 4-dioxane.

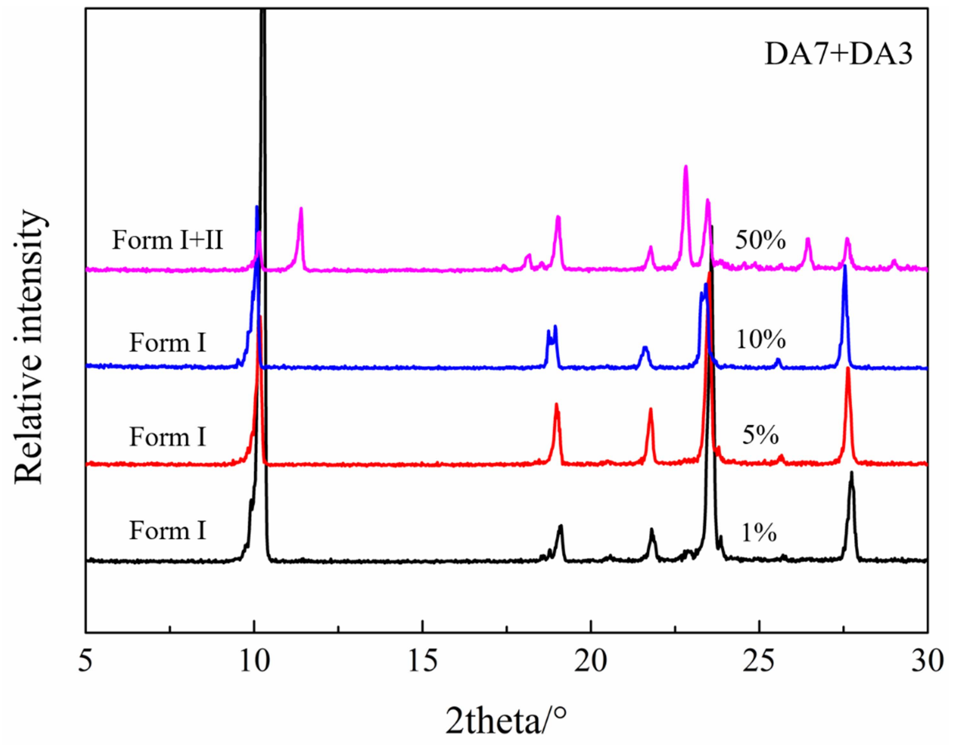

Figure S3 PXRD patterns of DA7 crystallized in presence of DA3 with different amounts in 1, 4-dioxane.

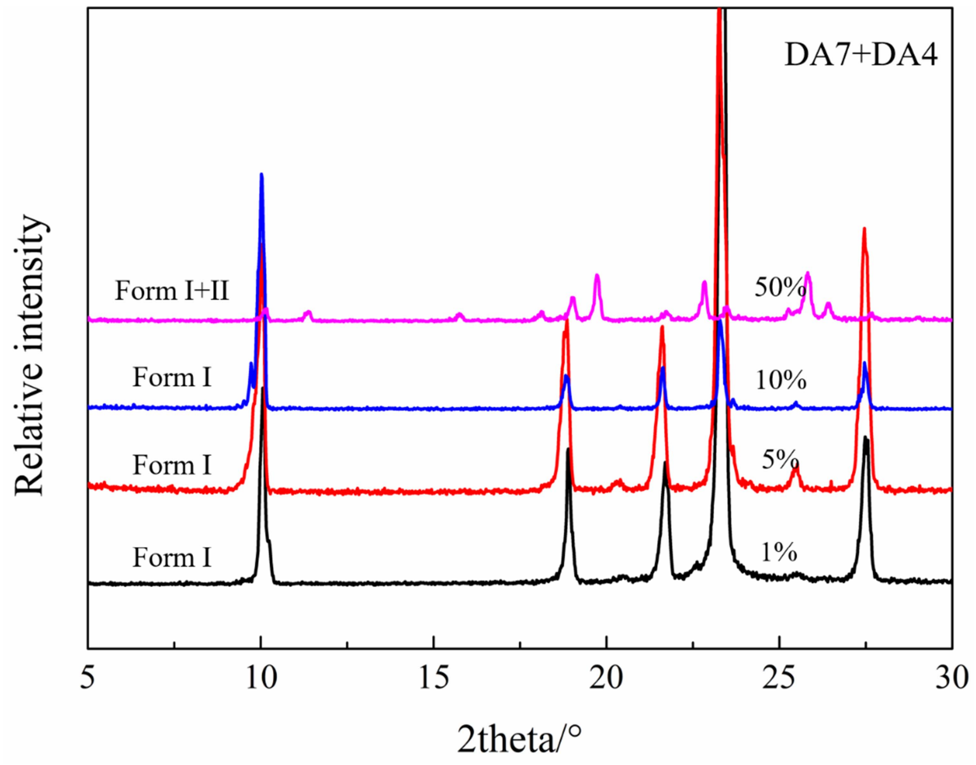

Figure S4 PXRD patterns of DA7 crystallized in presence of DA4 with different amounts in 1, 4-dioxane.

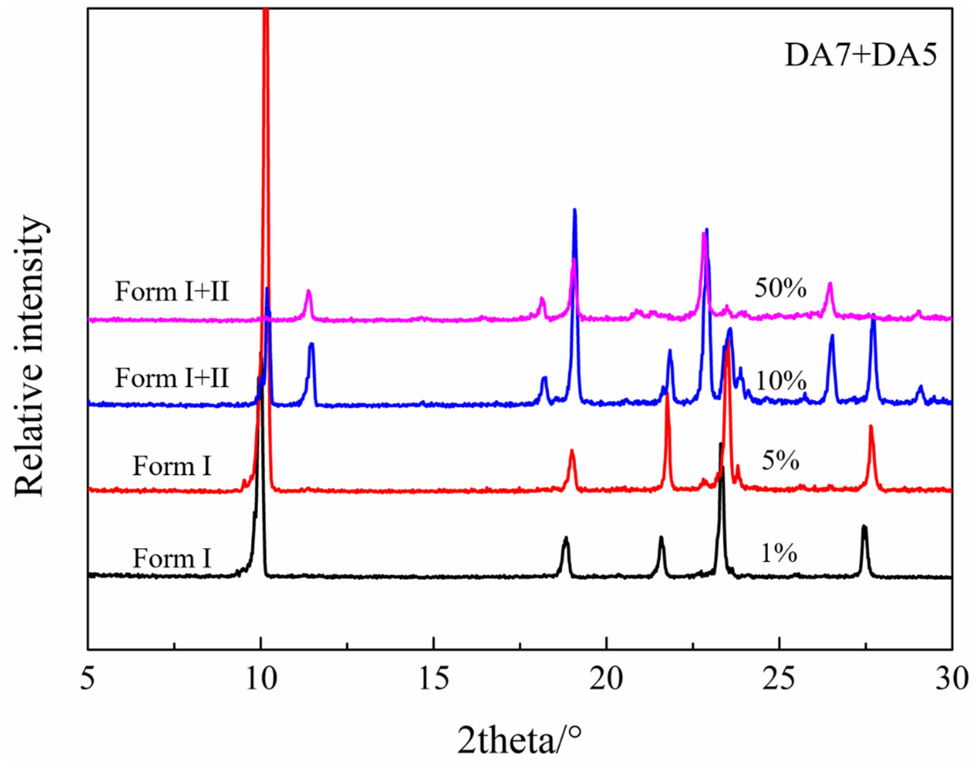

Figure S5 PXRD patterns of DA7 crystallized in presence of DA5 with different amounts in 1, 4-dioxane.

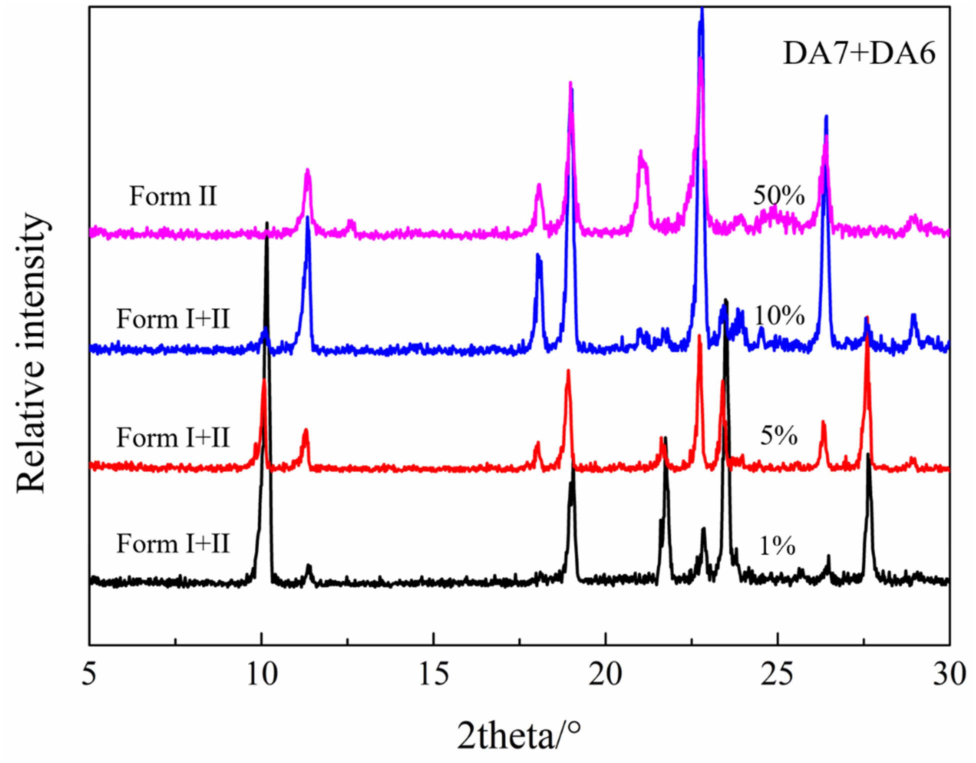

Figure S6 PXRD patterns of DA7 crystallized in presence of DA6 with different amounts in 1, 4-dioxane.

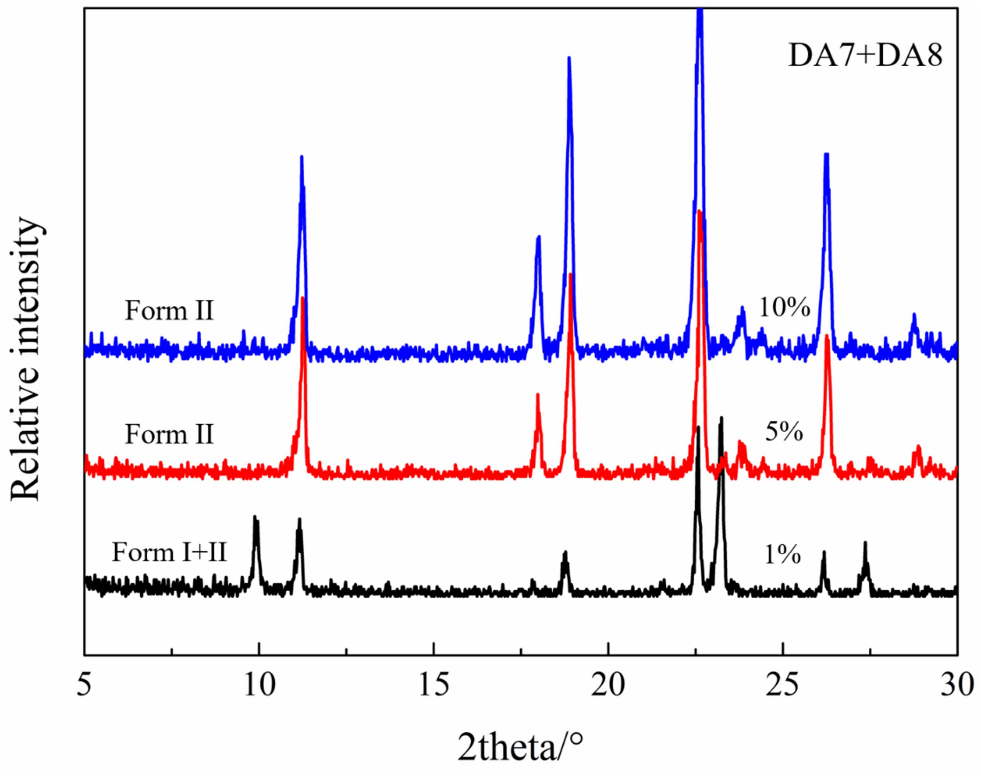

Figure S7 PXRD patterns of DA7 crystallized in presence of DA8 with different amounts in 1, 4-dioxane.

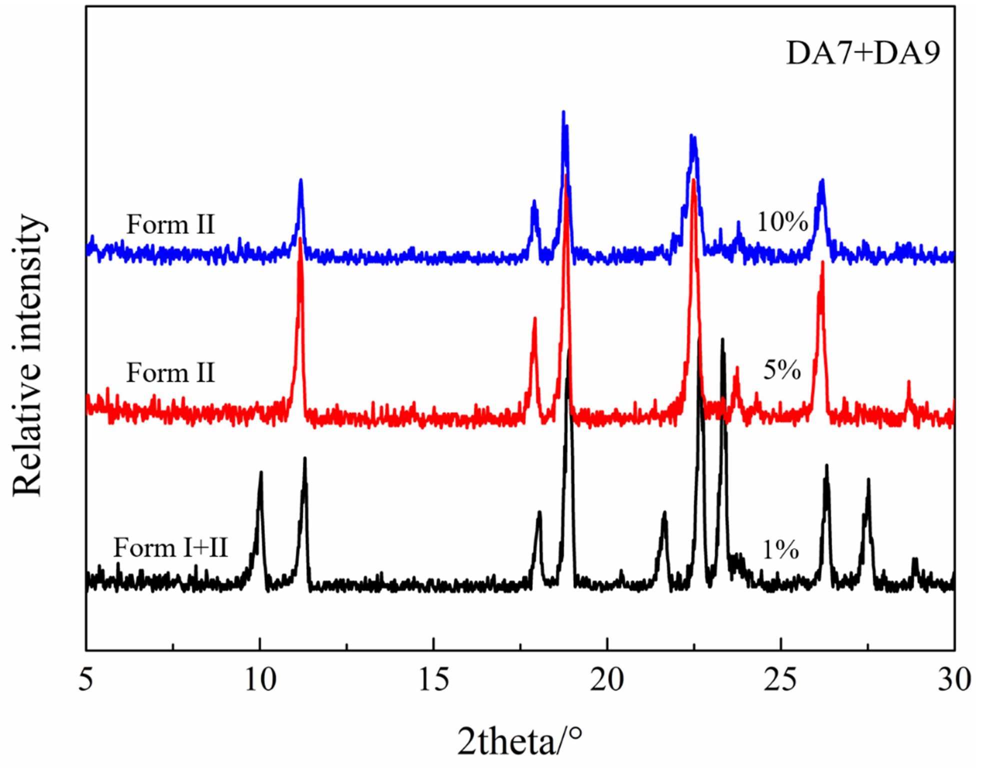

Figure S8 PXRD patterns of DA7 crystallized in presence of DA9 with different amounts in 1, 4-dioxane.

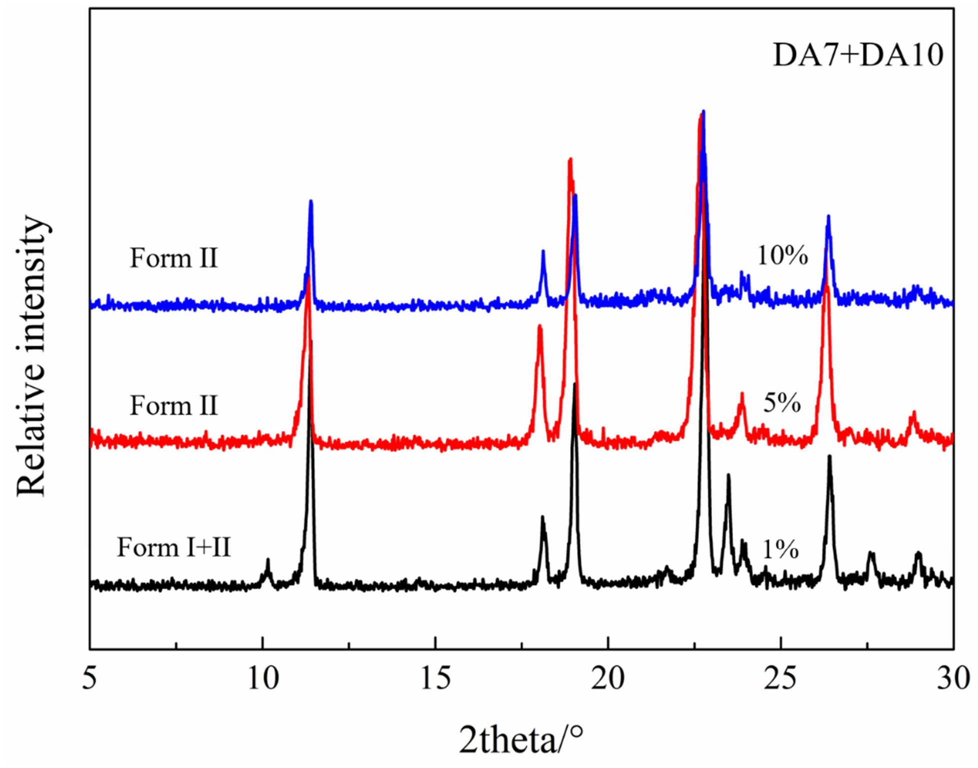

Figure S9 PXRD patterns of DA7 crystallized in presence of DA10 with different amounts in 1, 4-dioxane.

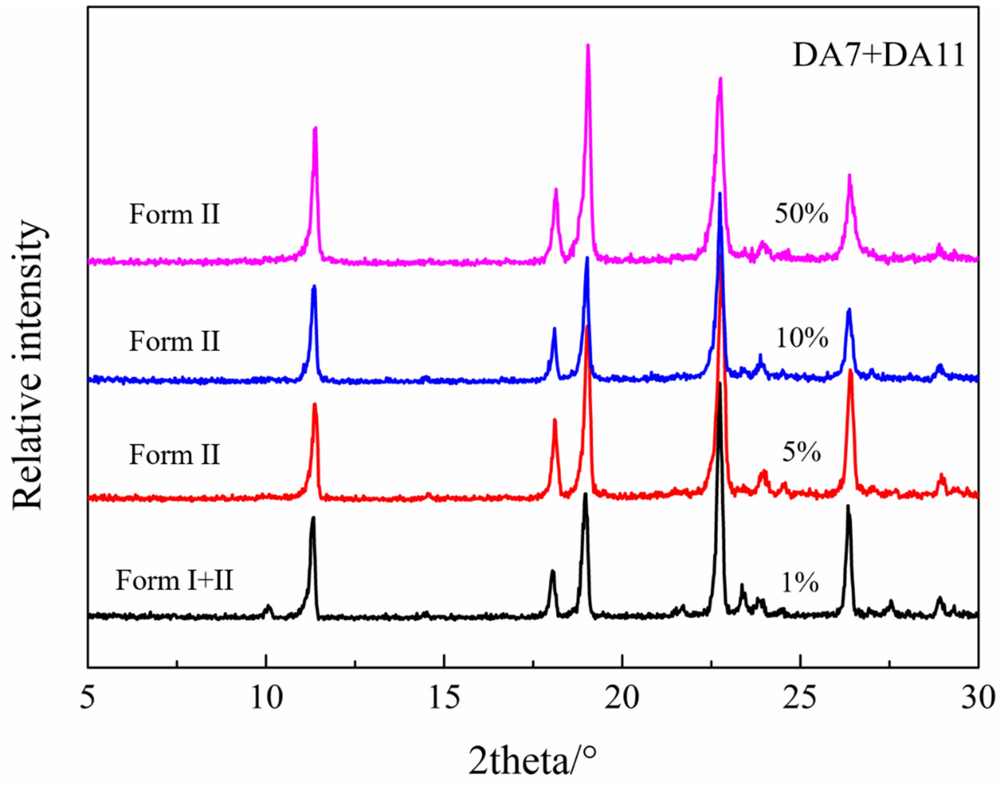

Figure S10PXRD patterns of DA7 crystallized in presence of DA11 with different amounts in 1, 4-dioxane.

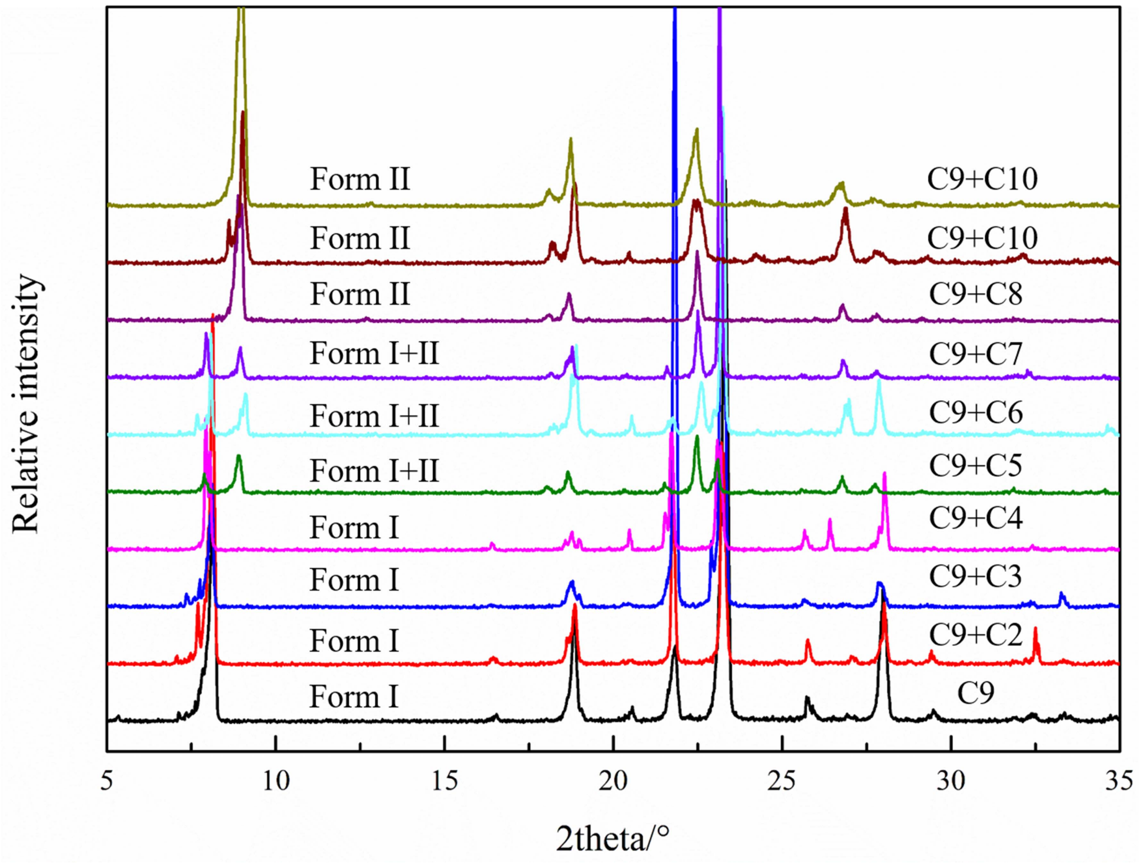

Figure S11PXRD patterns of DA9 crystallized in presence of additives at 1:10 mole ratio in 1, 4-dioxane.

S3. Solubility data of DA7 with additives in 1, 4-dioxane.

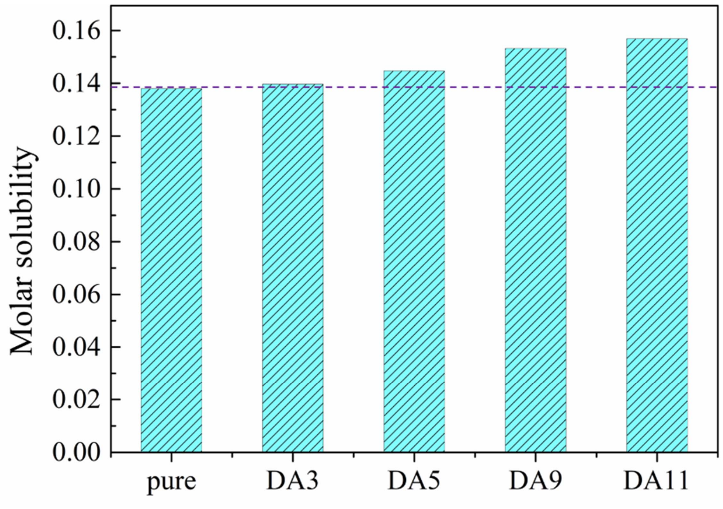

Figure S12 The molar solubility of DA7 with the concentration of 0.1464 mol additive / mol solvent in 1,4-dioxide at 298.15 K.

S4. Supplementary information for FTIR spectrum analysis

As shown in **Figure S12**, only the peak at about 1736 cm^{-1} is retained when DA7 in 1, 4-dioxane at low concentration, which should be the vibration band of non-hydrogen-bonded C=O groups due to the solvent with no hydrogen-bonding donors. With the concentration of DA7 increasing, a new band at about 1712 cm^{-1} appeared. And the higher the concentration, the higher intensity ratio of the band at 1712 cm^{-1} to that at 1736 cm^{-1}. It declares that the carboxyl groups of diacids self-assembly happens in 1, 4-dioxane after reaching to a certain concentration, and the degree of aggregation increases with the increase of concentration. It indicates that is intermolecular interaction of solutes rather than intramolecular interaction, *e.g.* molecule self-cyclization. It is of great difference from solvents with high HBD ability where the molecules exist as solvated or non-solvated monomers.

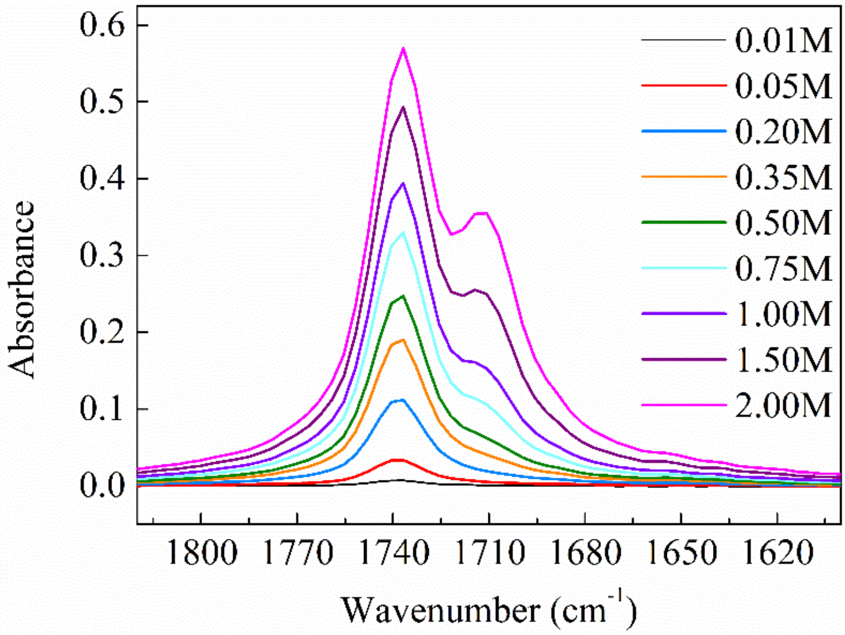

Figure S13 Solution IR spectra of pure DA7 over a concentration range in 1, 4-dioxane.

As shown in **Figure S13**, for DA2 and DA3 at both concentrations, there exist two vibration peaks of C=O, whose intensity ratio keep constant as concentration increases, respectively corresponding to free monomers (high wavenumber) and solvated monomers (low wavenumber). As additives, they also have a certain embodiment.

At the concentration of 0.25 mol solute/L solvent, there is no obvious signal of C=O aggregation for all diacids. When they work as additives, the peak intensity of solute aggregation at about 1712 cm^{-1} increases in all samples studied.

At the concentration of 0.6 mol solute/L solvent, there exists slight aggregation in DA6-11. However, when they work as additives, the peak intensity of solute aggregation increase obviously. More importantly, the peak positions of aggregation while adding additives DA8-DA11 are similar to DA7 solute added in the same amount, distinct from its own aggregation position (at higher wavenumber)

at that concentration. It suggests that these additive molecules indeed involved in DA7 solute self-assembly. What's more, as shown in **Figure S14** (2 cm^{-1}) and **Figure 5** (4 cm^{-1}), we found that the resolutions used had few effects on test results of chemical shifts, which indicated that the results were reliable.

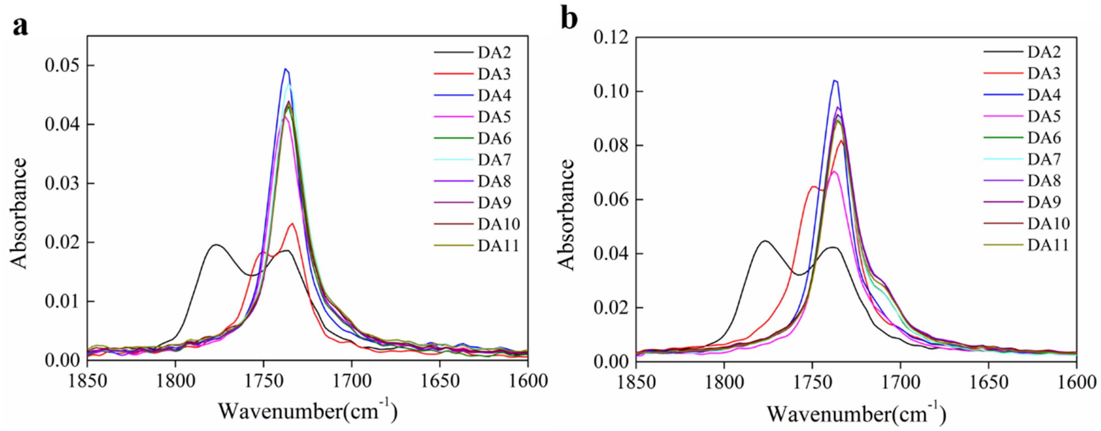

Figure S14 Solution IR spectra of pure additives in 1, 4-dioxane (a) at 0.25 mol solute/L solvent; (b) at 0.6 mol solute/L solvent.

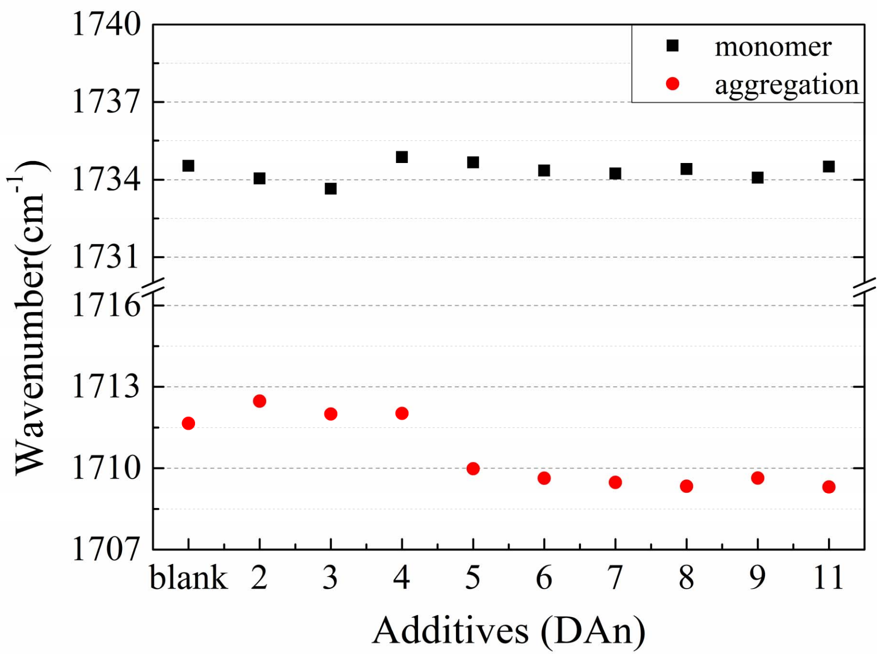

Figure S15 The solution IR vibration peak position of -C=O picked by using OMNIC software (resolution of 2cm^{-1}). DAn, n = number of C in molecule.

S5. The microphotographs of samples of DA7 obtained from dioxane with the present of additives.

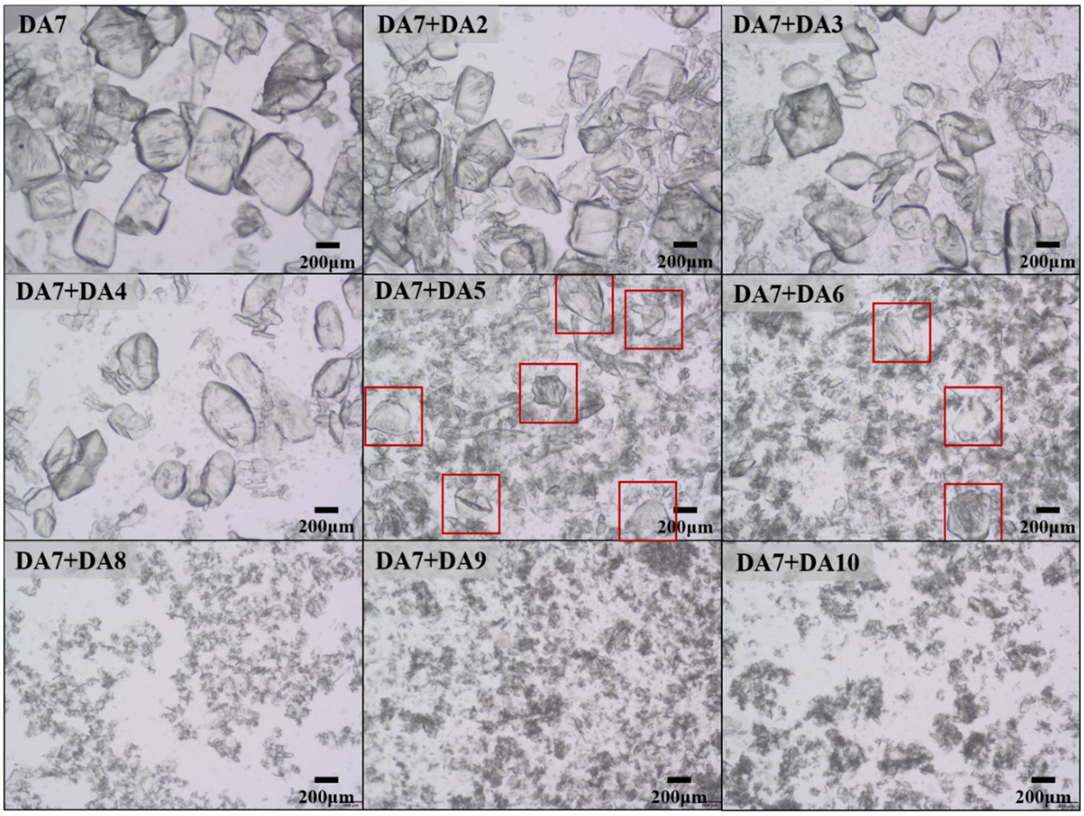

Figure S16 The microphotographs of samples of DA7 obtained from dioxane with the present of additives after cooling experiments. The samples used to observe crystal shapes were taken out after 20 min since nucleation before phase transition. Pure I crystal form obtained in dioxane is a bulk square plate. With the addition of DA2/3/4, the crystals have some very slight changes, tending to be diamond-shaped. When DA5/6 is added, it is obvious that a large number of small fusiform crystals appear and coalesce. It is worth noting that there is still mixed with bulk form I (in red boxes) (small fusiform crystals attached). The two crystal forms can be clearly distinguished. With the addition of longer carbon chain additives, only a large number of small fusiform crystals remain, which is form II. And there is no longer crystal form I, large crystals. It is obvious that the effect of additives on the growth modification of crystal form I is relatively weak. While adding DA5/6 that can induce form II, crystal form I nucleate concomitant with form II and still can keep growth similarly.

S6. ESP charges, ESP maps and binding eneries of DA7 and series additives

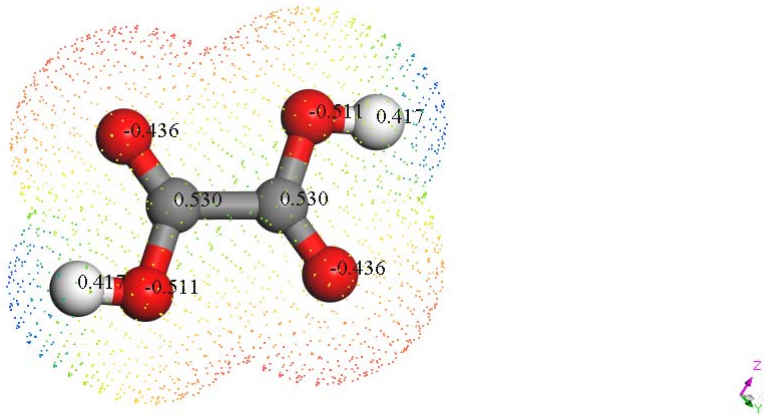

Figure S17ESP maps and ESP charges of DA2.

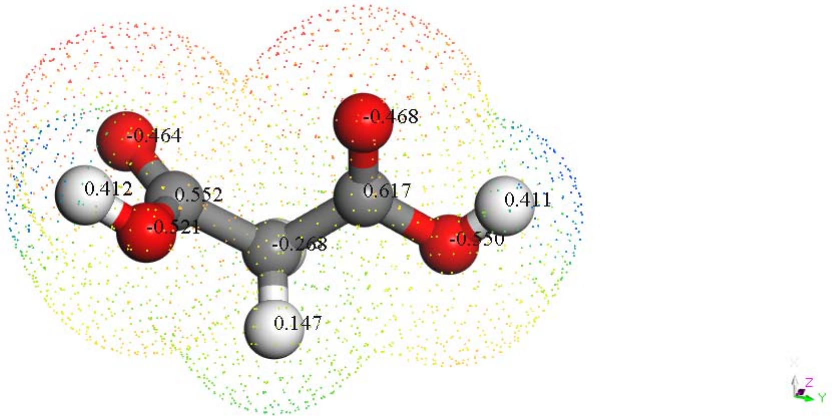

Figure S18ESP maps and ESP charges of DA3.

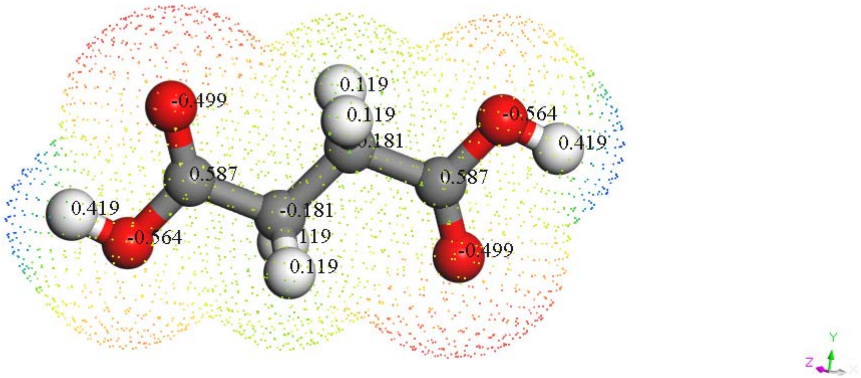

Figure S19ESP maps and ESP charges of DA4.

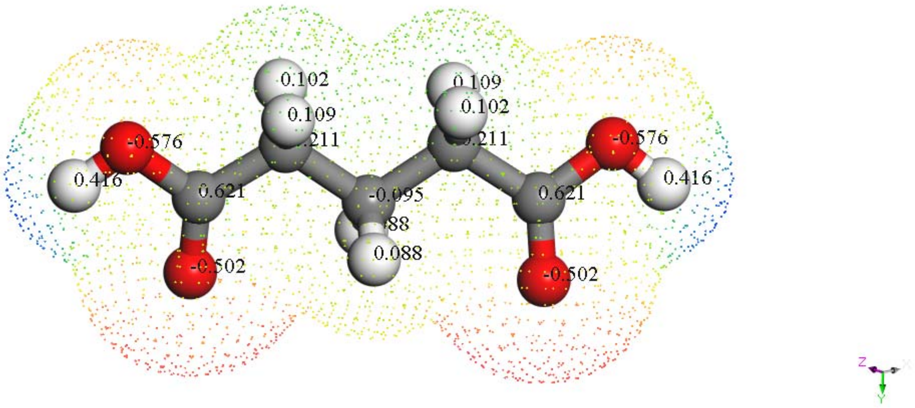

Figure S20 ESP maps and ESP charges of DA5.

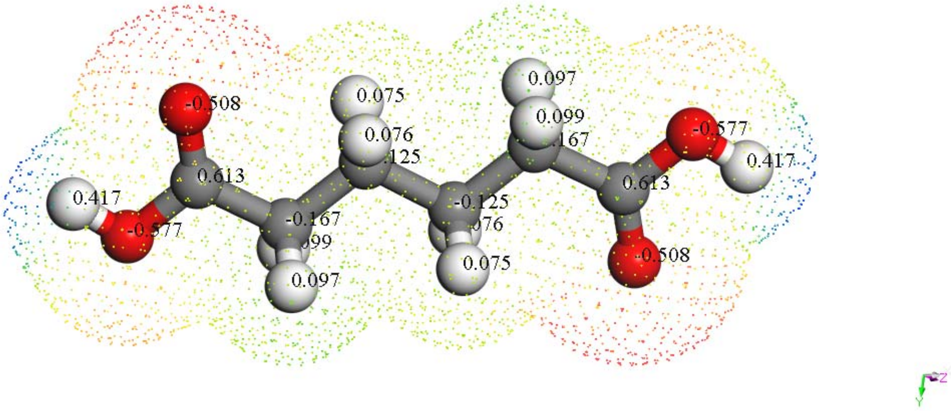

Figure S21 ESP maps and ESP charges of DA6.

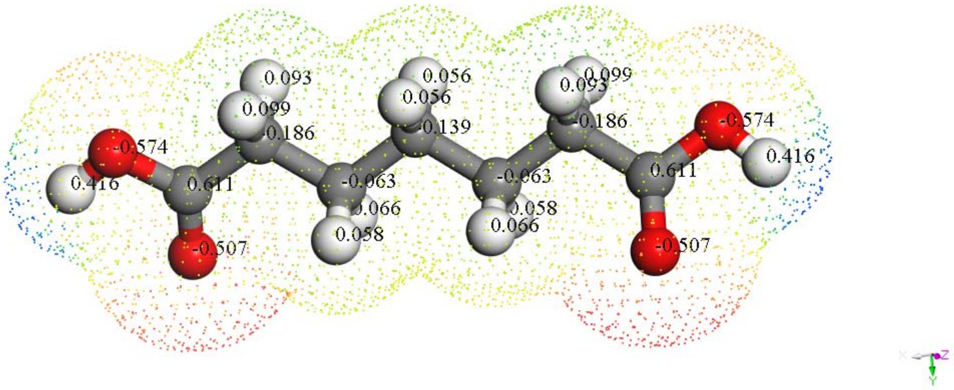

Figure S22 ESP maps and ESP charges of DA7.

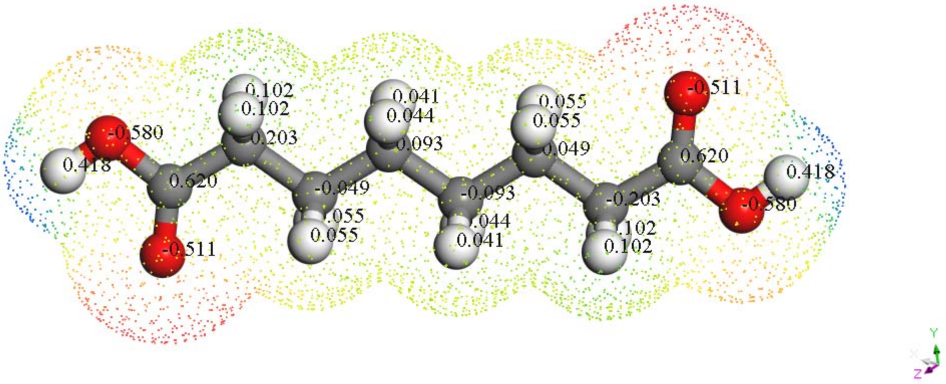

Figure S23ESP maps and ESP charges of DA8.

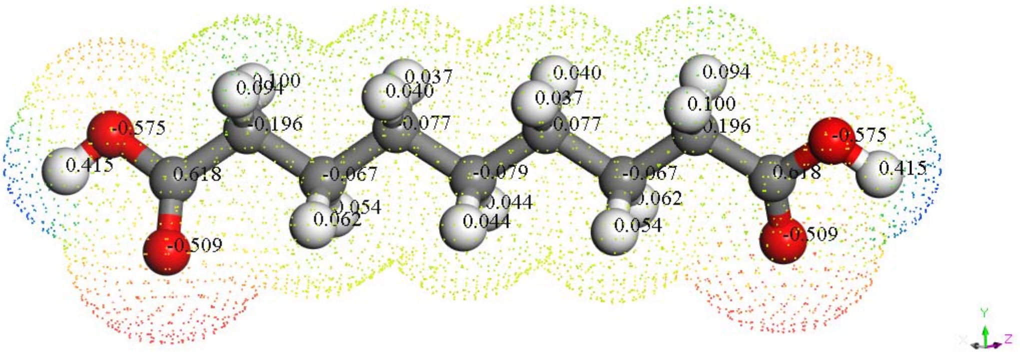

Figure S24ESP maps and ESP charges of DA9.

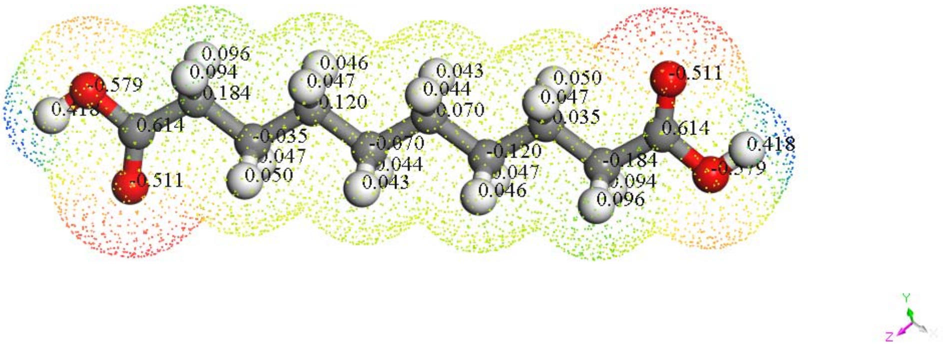

Figure S25ESP maps and ESP charges of DA10.

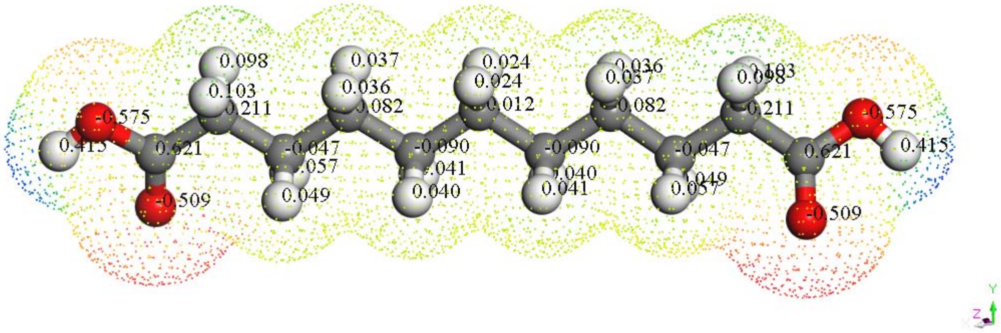

Figure S26ESP maps and ESP charges of DA11.

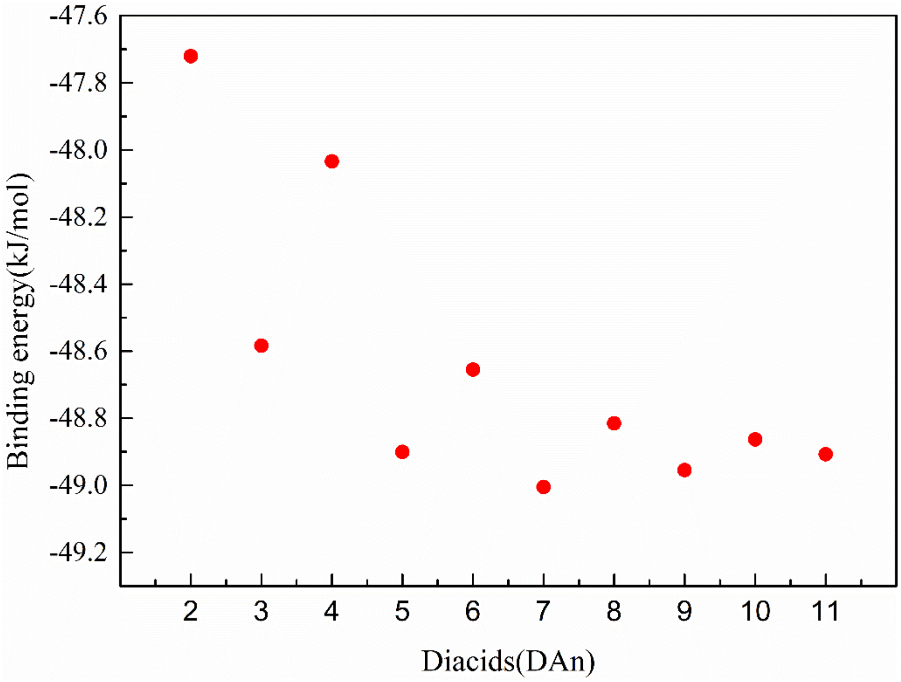

Figure S27 The binding energies for DA7-Diacids (DAn) hydrogen bonding cycle dimers in SMD solvation model (dioxane).

The results (Fig. S27) shows that the trend of solute-additive interactions is basically consistent with that of ESP charges though there exists a slight fluctuation. On the whole, compared with DA2-4, the stronger solute-additive interactions occur while longer carbon chain additives.

In fact, when a more complex molecule model is considered, there is always some gap with the actual situation, e.g. the interaction modes between carboxyls might be more abundant. In a sense, ESPs of individual molecules, as the fundamental factor of intermolecular

interactions, should give certain reference value for the interaction strength between those with other molecules.
